# The development of Liver Research Cymru, a new partnership to increase hepatology research activity in Wales

**DOI:** 10.3310/nihropenres.13663.1

**Published:** 2024-10-08

**Authors:** AD Yeoman, H Ahmed, A Akbari, K Cullen, A Davies, D Fitzsimmons, J Gao, K Hood, C Nollett, A Vincent, W Williams, TPI Pembroke

**Affiliations:** 1 Gwent Liver Unit, NHS Wales Aneurin Bevan University Health Board, Newport, Wales, NP20 2UB, UK; 2 Division of Population Medicine, Cardiff University, Cardiff, UK; 3 Population Data Science, Swansea University, Swansea, Wales, UK; 4 Swansea Centre for Health Economics, Swansea University, Swansea, Wales, UK; 5 PRIME Centre Wales, Cardiff University, Cardiff, Wales, UK; 6 Cardiff University Centre for Trials Research, Cardiff, Wales, UK; 7 Public contributor with liver experience, Wales, UK; 8 Cardiff Liver Unit, University Hospital of Wales, Cardiff, Wales, UK

**Keywords:** Early detection, Incidence, Prevalence, Cirrhosis, Deprivation, Mortality

## Abstract

**Background:**

The incidence and severity of liver disease in the United Kingdom have increased over the last 20 years. Many patients present with advanced disease with limited treatment options and subsequently high morbidity and mortality. There was also a significant correlation with deprivation.

Strategies that support the earlier detection of liver disease are paramount to reverse this trend. Despite significant progress in terms of novel pathways, the optimal strategy for early detection of liver disease remains unknown. Novel ways to tackle the deprivation gradient and reduce health inequalities are urgently required.

**Methods:**

Clinical research has an enormous role to play both in terms of identifying the true scale of this challenge, where current gaps exist, and to identify the optimal early detection strategies and their implementation. WE therefore established Liver Rsearch Cymru (LRC) a multi-disciplinary collaboration that seeks to maximise the benefits from our existing data sources and clinical networks and increase the output of hepatology research in Wales.

**Results:**

LRC has developed the first Wales wide research collaborative. We have successfully collaborated with the Secure Anonymised Information Linkage (SAIL) data resource to develop a greater understanding of liver disease burdens through comprehensive analysis of primary and secondary care data. We are now using this information to evaluate the effectiveness of local early detection pathways and to identify the scale of delays in diagnosis with a view to addressing this important care gap.

**Conclusion:**

LRC has successfully brought together patients. Hepatologists and population/primary care academics to better understand current discrepancies in the early diagnosis of liver disease in Wales. In addition, it has laid a foundation for future research work based both on our preliminary findings and allowed us to collaborate with other more established liver disease research groups.

## Plain Language Summary

Liver Research Cymru is a partnership, funded by the NIHR to support research into ways of understanding the current burden of liver disease in Wales, its relationship to poverty and to use this information as a way of asking more intelligent and practical research questions in the future. This is all being done to support liver disease being diagnosed earlier so that treatment and support can be provided earlier to reduce the harm to patients.

LRC chose to do this in 3 programmes of work. Firstly, the partnership brought together liver experts across Wales with health care researchers with expertise in primary care and population health and identified a group of people with lived experience of liver disease to make the work relevant to patients needs. Secondly, we explored data sets held within Wales to identify, at a high level as many people as possible known to have liver disease in Wales and realte that to areas of deprivation (poverty).

Finally, LRC is using the knowledge gained from the first 2 programmes to design future research work and to set up additional research partnerships with other NIHR funded research groups.

## Introduction

Wales have a high burden of liver disease ^1^, but historically, there are few established liver services. This was a major driver for change, leading the Welsh Government to commission a national strategy, the Liver Disease Delivery Plan, which was to be overseen by the Liver Disease Implementation Group (LDIG) in early 2014 and launched in late 2015.

( https://www.gov.wales/sites/default/files/publications/2018-12/liver-disease-delivery-plan-2015-to-2020.pdf. The delivery of high-quality research was one of the six key aspects of the plan as follows:

1) Prevention
2) Early detection
3) Fast and effective care
4) Improving Information
5) Living with liver disease
6) Improving research

Success has been achieved in several of these domains, including improvements in the early detection of liver disease through the development of a national abnormal liver blood test, significant increase in the number of clinical hepatologists, and high-quality secondary care data via a liver disease registry.

However, there has been no significant progress in this research domain. This lack of progress was almost entirely due to the lack of funded liver clinical research posts or sessional consultation time dedicated to liver research within Wales.

Therefore, the National Institute of Healthcare Research (NIHR) called to support the development of new research partnerships in liver disease at an opportune time to address this deficiency and build on the existing successes in early detection made via the LDIG.

## Methods

### Patient and Public Involvement

Two people (Williams, Vincent) with lived experience were known to the clinical hepatologists through engagement work part of the LDIG. These two individuals were invited to be involved from the very beginning of the partnership and helped draft the grant submission to the NIHR. Therefore, they were integral to the original submission and listed as co-applicants as a consequence. Upon being successful in obtaining funding the British Liver Trust were also engaged to assist in the recruitment of the broader PPI team. This group was provided with support by the PPI lead (Nollett) in the lead up to initiation of the project and as part of work programme 1. The PPI team was also regularly updated by the co-lead applicants as to progress on the SAIL data extraction with a particular focus on issues arising from their own experience of receiving a liver disease diagnosis. This aspect in particular drove the intention to evaluate the impact of an existing programme to improve early detection and also another analysis exploring time to diagnosis from first abnormal liver blood tests. Furthermore, the PPI group was presented with the results that have fromed the first paper from the SAIL analysis.

Following the launch of the NIHR Liver Disease Research Partnership call, a working group was formed, titled Liver Research Cymru. Three work programs were described in the original proposal.

1) **Building the research community**
2) **Connecting and understanding the data**
3) **Prioritising and designing studies**

### Building the research community

As there was no established liver disease research community in Wales, there was a need to establish a partnership network of healthcare professionals, researchers, and the public across Wales. Clinical partners included consultant hepatologists, general practitioners (GPs), microbiologists/blood-borne virus (BBV) teams, public health consultants, and allied health care practitioners, including hepatology and BBV specialist nurses. Representation was sought from all the Welsh health boards. Academic partners were brought together from the Centre for Trials Research and the Division of Population Medicine at Cardiff University, and Swansea Centre for Health Economics, Population Data Science, and the Secure Anonymised Information Linkage (SAIL) Databank at Swansea University. The benefits of this were two-fold: first, it brought experience of undertaking population health research in the Welsh context, which would strengthen the project, and second, it provided mentorship for the clinicians within the research partnership.

The partnership's initial focus was to strengthen the links made via the work of the LDIG and use them to build the additional necessary links to lead research focused on early diagnosis, identifying inequalities, and evaluating the impact of deprivation.

The project management group met monthly, laying the foundation for the network model. The broader stakeholder group contributed to data review and research synthesis, and individuals contributed to the focused elements of project development. The partnership was therefore connected with successful research teams that have addressed similar priorities for other diseases, such as the screening, diagnosis, and early prevention of the cancer research team at the Division of Population Medicine, Cardiff University.

Mentorship was provided by the senior research team (Hood, Fitzsimmons) to liver disease specialists (Yeoman, Pembroke) with the additional view of recognizing training and upskilling needs. Network management was facilitated by a project manager from within the lead applicant’s organization (Aneurin Bevan University Health Board (ABUHB)).

In addition to developing a research partnership within Wales, Liver Research Cymru aimed to build and strengthen relationships with existing research groups active in this field across the UK. In this regard, one of the co-lead applicants (ADY) was already a member of the British Society of Gastroenterology (BSG)/British Association for the Study of the Liver (BASL) Research Development Group and also co-chairs an informal special interest group (SiG) on the Early Detection of Liver Disease in the UK, which constitutes an excellent opportunity for ongoing external partnerships and information dissemination.

The informal SiG came out of a desire to share best practices across the UK and help set the direction of future work with regard to clinical pathways and research. Tangible outputs from the group comprised of two publications. The first was the principles and methods of early detection of liver disease ^2^, and the second was a systematic review of existing pathways in the United Kingdom ^3^.

The SiG comprises membership from each of the four nations within the UK and is represented by the key clinical research groups currently working in the field of early detection of liver disease (Dundee, Leeds, Royal Free and University College London, Portsmouth, Nottingham, and Newcastle). As such, there is an established collaborative network available for Liver Research Cymru to build upon and develop stronger relationships and partnerships.

Patient and Public Involvement (PPI) is an integral part of the network model and is included to contextualize findings from the data from a patient/service-user perspective, prioritize and develop research questions, input into grant applications, and drive participation in liver disease research. To expand the initial patient representation, a public advisory group will be established consisting of eight people with a personal experience of liver disease. The aim was to encompass representation from every health board in Wales, those of working age, underserved groups, and the predominant etiologies of liver disease in Wales.

Patient and public involvement was already a function of the work of the LDIG, and we invited two people with lived experience via the program of work to form a core project group to develop the initial grant proposal. The LDIG also had formal links with the British Liver Trust, which was also consulted and instrumental in the grant development proposal stage.

### Planned outputs from work programme 1

**Figure 1.**
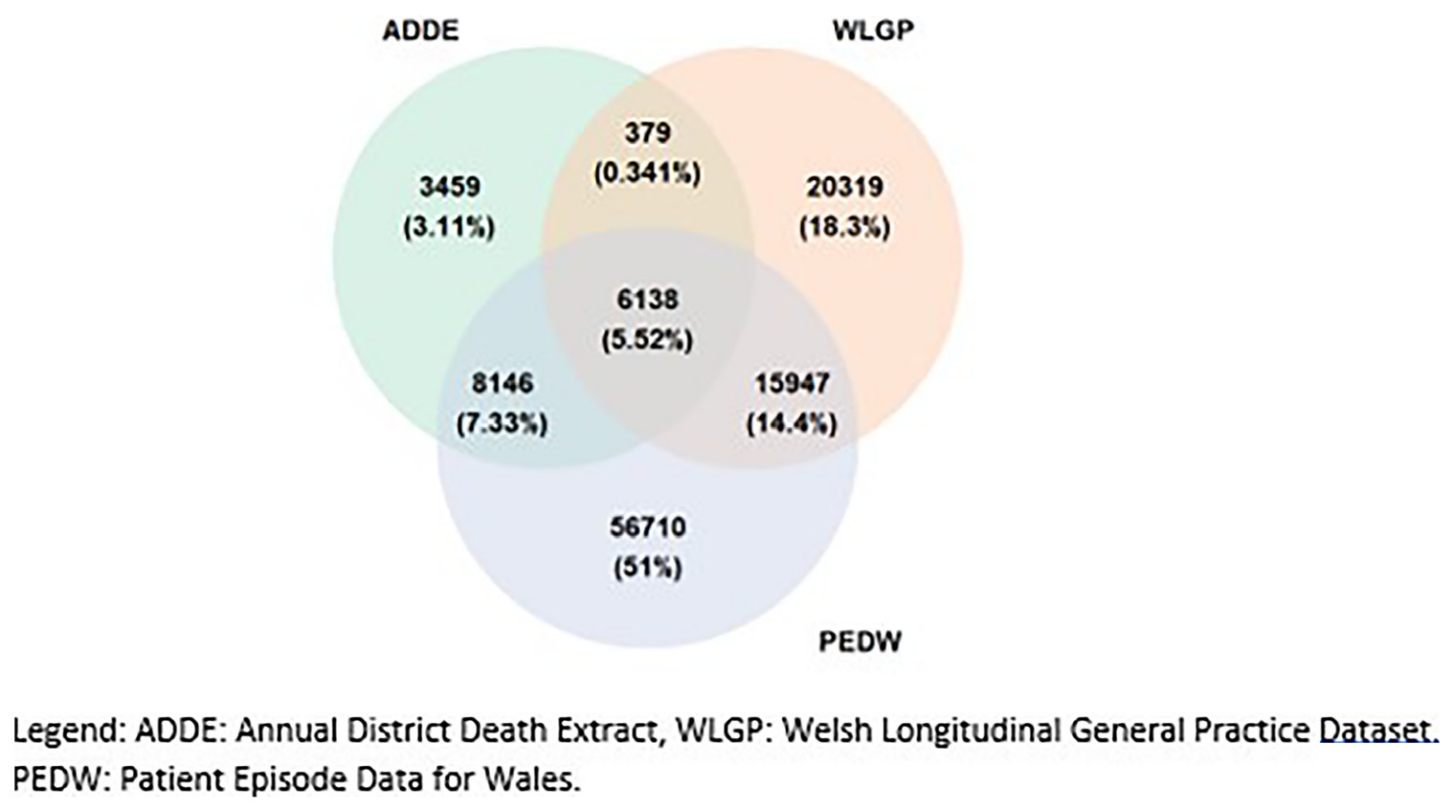
Composition of the study cohort based on PEDW (secondary care), WLGP (primary care), and ADDE (death certification) data sources.

The Liver Research Cymru network aims to develop an inclusive and cohesive group within 6-months to review the data generated in WP2 and contribute to the design and prioritization of studies leading to NIHR Phase 2 bids in Work Programme 3 ( Figure 1). In addition, we set out to utilize this network to springboard a bid to establish this network as a health and care research unit as a medium-term ambition.

### Connecting and understanding the data

As already mentioned, the LDIG created The Wales Liver Disease Registry, which provided a rich source of data on changing patterns and burdens of liver disease in Wales from 1999 onwards.

The methodology of this registry was based on the International Classification of Diseases (ICD) version 10 (ICD-10) code mapping in the HepaHealth project (available at https://easl.eu/publication/hepahealth-project-report/) and the 2021 expert panel consensus ^4, 5^ (Pimpin *et al.*, 2018, Hagström *et al.*, 2021). Additionally, codes reflecting the natural history of liver disease were defined to enable monitoring of disease progression over time from the underlying etiology (i.e., alcohol-related liver disease, hepatitis C infection) to cirrhosis, portal hypertension, decompensation, liver cancer, and death. ICD-10 mapping to read codes has been completed, thereby providing high-level health intelligence on changing patterns of the incidence and prevalence of liver disease in Wales ^1^. However, there were limitations to this approach as it was entirely dependent on hospital-based coding.

The Secure Anonymized Information Linkage (SAIL) Databank is one of the richest anonymized individual-level, population-scale trusted research environments (TRE) in the world, and contains a variety of health and social care, administrative, geo-environmental, and specialist data sources (saildatabank.com, Ford *et al.*, 2009, Rodgers *et al.*, 2012).

SAIL contains 86% primary care and 100% secondary care data for all Welsh residents and those receiving NHS Wales services. The wealth of information available in the SAIL Databank includes lifestyle and clinical data (alcohol excess, BMI, injecting drug use, smoking, and diabetes), prescribing data, clinical results extracted directly from laboratory reporting systems (e.g., abnormal liver function tests and thrombocytopenia), and environmental and socio-economic deprivation data using Lower-layer Super Output Area (LSOA) linkage. In addition to diagnostic, treatment, and outcome data, demographic, socioeconomic, operative codes, and pathology data will be included in the data.

This work programme constituted by far the largest proposed body of work within the partnership and correspondingly was allocated the greatest proportion of the budget.

The Liver Disease Registry methodology was applied to the SAIL Databank via the development of a specific protocol, thus enabling the capture of outpatient diagnoses of liver diseases at an earlier stage of presentation (Ratib *et al.*, 2014). Discrete pre-diagnosis stages of i) liver risk factors, alcohol, obesity/metabolic, and injecting drug use, and ii) routine blood test abnormalities will be defined. The coding methodology will be reviewed as it is integrated with primary care data. Further adjustments will be made to ensure complete capture of all available and relevant read codes, which are, in essence, a thesaurus of clinical terms. Further information on read codes and their application in clinical practice in the NHS can be found at https://digital.nhs.uk/service/terminology-and-classifications/read-codes). Furthermore, associated risk factors and clinical investigations will also be brought into the research ready data asset (RRDA).

We aimed to test the scope and utility of RRDA to address three key objectives.

1) Describe key clinical and socio-demographic features associated with liver disease diagnosis, later stage presentation, mortality on first hospital admission and disease progression.

Descriptive logistic regression models will investigate a number of potential explanatory variables, including (but not limited to) social deprivation and difficult-to-reach populations. These can be defined as difficult due to geographic locality (including coastal regions, post-industrial towns, and inner cities as well as remote rural locations), language or cultural barriers, or stigma, especially seen among marginalized groups. The description of features associated with the development of compensated advanced chronic liver disease (cACLD) is of particular interest. These patients are a key group for early diagnosis in primary care, and these analyses will improve our understanding of disease progression (including HCC development) and potentially lead to future research on appropriate screening strategies. Overall, these descriptive analyses will help us to better understand who is at a greater risk of liver disease and has poorer outcomes from liver disease. Furthermore, this work will inform subsequent funding applications to allow targeting of the population most at need.

2) Assess the ability to evaluate clinical pathways

We developed a data platform that enables rapid evaluation and direct information on the subsequent improvement of service changes and associated future health economic implications. We aimed to test and refine our ability to do this by evaluating the All-Wales Abnormal Liver Blood Test Pathway, launched in October 2021. This pathway was implemented in a staggered approach across Wales, which allowed us to analyze the intervention in comparator groups using data held within the SAIL Databank across University Health Boards in Wales. This analysis included both the clinical and cost-effectiveness outcomes. Following this work, we aimed to develop reproducible pipelines to enable rapid clinical and economic analyses of future pathways, not just in Wales, but across the UK, using routinely collected data and SAIL as the trusted research environment (Innes *et al.*, 2022, Petta *et al.*, 2021).

3) Develop frameworks to assess time to diagnosis and disease progression

Linked primary care, secondary care, laboratory, pathology, and mortality data may allow us to describe the clinical encounters from a first presentation with a liver-related symptom to the first liver disease diagnosis and assess the potential for earlier diagnosis and treatment, as well as resource use and associated costs. The main objectives of this work will be descriptive, aiming to better understand the data, including the size, complexity, and value of the repeated measures. Furthermore, we aimed to draw on expertise from other disease areas to define parameters of interest (e.g., code lists of agreed liver-related symptoms and the period that most accurately reflects time to diagnosis). The overall aim of this work is to complete the necessary pilot and feasibility studies to prepare us to apply for funding for subsequent early diagnosis research, which will use causal inference methods to fully and robustly understand pathways to diagnosis and treatment and identify areas for intervention.

Following this work, the patient and public involvement group will help interpret the findings from the data integration and provide their own perspectives (from their lived experiences) on the liver journey, including missed opportunities for diagnosis.

This data analysis will enhance our understanding of the natural history and burden of liver disease in males. This workstream will develop methodologies to synthesize high-quality data, underpinning future work in the timeframe of the NIHR Phase 2 funding call. In particular, sample size, potential bias, and approaches to mitigate bias will be identified, as well as opportunities to utilize routine data to provide outcome data for prospective studies. We aimed to develop liver disease progression and features of mortality data relevant to under-researched areas, including palliative care and networks for advanced complex liver disease outside the liver transplant setting. Approaches to identifying the risk factors for missed opportunities in diagnosis will be implemented.

Therefore, it is anticipated that this analysis of liver health in Wales will address the gaps in service provision and needs for service development, utilizing research methodology to identify the most appropriate methods. These potentially low-hanging opportunities to bring about rapid and high-impact service development will be implemented through the existing LDIG network and the impact will be assessed through the Liver Research Cymru partnership.

### Prioritising and designing studies

Following the initial establishment of the Liver Research Cymru partnership and the development of the liver disease RRDA, a specific workstream will convene to identify specific research questions that can be enabled using the RRDA, and how it could be potentially expanded and iteratively developed as new priorities are identified. The priority of these questions and study design will reflect the newly formed partnerships within Wales and the clinical needs established in the Liver Disease Implementation Group with patient advisory group input. The key goal is to develop a research application in the NIHR Phase 2 call, either as a standalone or as part of a wider partnership. It is anticipated that the primary focus of this research will reflect the need to improve the early diagnosis and screening of liver diseases in Wales. Complementary research questions will incorporate i) modelling of the services required to meet the growing incidence of liver disease, including assessment of cost-effectiveness, and ii) identification and engagement with under-served groups at increased risk of liver disease.

This work package will facilitate research prioritization among partners and external stakeholders. Initial long lists of research priorities will be identified from James Lind Alliance Priority Setting Partnership Reports, The National Institute for Health and Care Excellence (NICE) clinical guideline research recommendations, national Special Interest Groups (SiGs), and national liver disease delivery plans. They will be considered in the context of data insights from Work Package 2 regarding burden and need. Liver Research Cymru will also prioritize working with other groups funded by this call to deliver appropriate research activity within Wales to maximize recruitment.

The public advisory group will hold a third meeting to prioritize research questions from a patient/caregiver perspective and reference the long lists identified. A fourth meeting of the public advisory group will be held to gather patient/caregiver input into the design of the proposed research and the final grant application.

Prioritized research will be designed with methodological input from the Centre for Trials Research, Centre for Health Economics, Division of Population Medicine, Population Data Science Group, Swansea University, and external experts, as required. WP leads will link with existing health and care research wholesale infrastructure, such as the Research Support & Delivery Service, to design decentralized studies that minimize the burden on participants and the NHS. This builds on our experiences supporting the UK-wide PANORAMIC trial, which recruited and followed up 25,000 people (1,800 in Wales) from primary care in 5-months without face-to-face contact. In the design of future studies, we will use our links with the Population Data Science Group and the SAIL Databank, and our substantial experience of using routine health data (including NHS England, Clinical Practice Research Datalink (CPRD), QResearch, etc.) to maximize the capture of participant information, including patient-reported outcome measures, and follow-up data from routine records (including data for health economics), using SAIL as a dedicated TRE for studies that recruit participants from outside Wales.

Therefore, Liver Research Cymru aims to develop a new research network combining clinical hepatology experience with established research expertise in Wales. The application and development of the novel Liver Registry methodology within the SAIL Databank will provide a unique liver disease RRDA encompassing risk factors for primary and secondary care diagnoses, prescribing, laboratory, geo-environmental, and mortality data. Methodology and preliminary data review by a broad range of methodological experts will inform the development of research projects as part of NIHR Phase 2 bids. Further expansion to acquire and use UK-wide data will be explored, with the SAIL Databank TRE being able to store and access anonymized data from anywhere in the world. This project will provide important epidemiological data relevant to the development of hepatology services in Wales and across the UK in the evolving post-COVID-19 environment.

## Outcomes

### Work Package 1: Building the research community

Two patients living with liver disease in Wales were members of the core project team and involved in developing the grant application. Both are experienced patient representatives: (WillaimsWW) is a member of the established Liver Disease Implementation Group (LDIG) living in the Cwm Taf Morgannwg (CTM) Health Board area, and the other (VincentAV) is a member of the Obesity Empowerment Network and lives in the Aneurin Bevan University (ABUHB) Health Board area.

An important aspect of WP1 is to facilitate the involvement of patients in the research community. WW and AV advised that this would be best achieved with a dedicated Patient Advisory Group (PAG), led by themselves, which could feed into the main project team meetings, share their lived experiences with the clinical team, and contribute to developing future funding calls.

The British Liver Trust was instrumental in helping to recruit six other patients to join the PAG via their extensive network and a Wales-based officer. They advertised the opportunity through regular groups and social media channels. Our aim was to involve at least one patient from each of the seven health boards in Wales to enable reflection on care in each area. In line with the UK Standards for Public Involvement, we also considered diversity within the group, including men, women, and individuals with different liver-related conditions. Our final group of eight provided representations from each health board and consisted of four men and four women with a range of conditions, including cirrhosis, Primary Biliary Cholangitis (PC), Non-Fatty Liver disease (NAFLD), hemochromatosis, autoimmune hepatitis (AIH), and alcohol-related liver disease.

The PPI lead (Nollett) conducted an individual telephone induction with each PAG member, which covered their role, mutual expectations, payments, and support and learning needs. Members were signed to the free Health and Care Research Wales training on public involvement in research and offered payment for their time at the recommended rate of £25/hr. The individual meetings provided an opportunity to establish rapport with the patient and public lead and share their personal stories in a one-to-one setting before sharing with the group.

Two PAG meetings were held. These were conducted online to facilitate inclusivity and enable easy access for members from a wide geographical area with chronic conditions that might make travel difficult. They were facilitated by the PAG lead (Nollett) and attended by the project leads (Yeoman & Pembroke). The first PAG, held in month three of the project (June 2023), was an opportunity to introduce group members and establish working relationships. The project leads (Yeoman, Pembroke) presented a background to the project and liver disease research in Wales, and members were able to share their lived experience with the project team. They were reminded of the support provided by the British Liver Trust.

The second PAG meeting was held in month nine of the project (December 2023). The project leads spoke about the data expected from the SAIL resource and asked the group to provide feedback on the list of comorbidities and laboratory tests that might indicate risk factors for liver disease. In particular, individuals talked about their mental health in relation to liver disease, and how this can be both a result of, and a contributor to, liver disease, and should be considered as a trigger for identifying liver disease (e.g., obsessive compulsive disorder leading to alcohol use leading to liver disease). This was enlightening for the project team and considered an important avenue for further research. Individuals mentioned problems such as depression, anxiety, insomnia, and fatigue. They also reported experiencing stigma and shame both from their communities and from healthcare professionals, who also discussed the liver disease-related questions being distributed through Health Wise Wales and the potential for research partnerships with the University of Bournemouth.

Health Wise Wales is an online platform ( http://healthwisewales.org) that helps connect researchers with over 40,000 registered participants in Wales to help answer specific questions in relation to their health on a given subject. A project was developed using LDIG funding to generate a series of questionnaires.

1) What do I know about liver disease?
2) My risk of getting liver disease
3) How does having liver disease make you feel
4) Stigma of liver disease

The survey period has recently been closed, and approximately 2,200 responses have been generated. Once the process of analysis of this data has been concluded, the intention is to incorporate the findings into WP3 activities.

PPI lead members (Vincent, Williams) attended monthly project team meetings to provide feedback from the PAG group and provide an ongoing patient perspective in project planning.

Two unplanned activities were developed as a result of feedback from the PAG meetings and led by the WW and AV. The first was an informal meeting of the PAG held in month 6 (September 2023) with the aim of maintaining the group connection and providing a space to meet and discuss life with liver disease without the project team present. The second was a survey of the PAG members, developed by AV and WW, about the patient journey and quality of life, which led to discussions about mental health in the second meeting.

Owing to delays in refining the clinical methodology and the results from the study data from the SAIL Databank, the full final results of the methodology could not be presented to the PPI group prior to the end of the funding period. However, since the initial work has been completed on the methodology and results showing the RRDA cohort and demographic and clinical characteristics, a final online event is planned for the summer of 2024 to disseminate the results from the SAIL methodology and RRDA, seek further feedback, and maintain an ongoing connection between clinical researchers and the PAG to assist with potential future research projects.

The project team presented a poster at the Southwest Society for Academic Primary Care conference in April, 2024. The aim of attending and presenting the poster in the conference was to highlight the work that the group had done and to look to develop links with primary care-based researchers across the UK. One member of the PAG was supported to present the poster jointly with a member of the research team. He reflected: “ *There was a steady flow of people taking an interest in the poster during the lunch and afternoon break. And I was glad of the opportunity to speak about my own experience, how early detection by my own doctor within primary care led to my being alive today. Proof that early detection of liver disease is crucially important in preventing this growing problem of liver disease. Also, being in attendance at the conference was a real eye opener for me as a patient, to see how much research is being carried out on behalf of people like myself. Also, the incredible pressures and strain that is put on primary care. It made me more appreciative of the work that is being done in research*.”

The presented poster can be found in the supplementary materials.

At the end of the project, the PAG members were invited to provide anonymous feedback on their involvement. One member stated that more public involvement is required in liver disease research. They suggested involving medical staff with the conditions under study. Another member reported finding it difficult to contribute to the meetings and was unsure what they could add or say to the clinical team. They suggested having fewer presentations per meeting and providing more information in advance. This feedback will be considered and changes made when PPI involvement is required for future projects.

### Work Programme 2: Connecting and understanding the data

The Liver Research Cymru partnership aimed to develop an RRDA capitalizing on the previous Liver Disease Registry experience in Wales. In this study, we aimed to describe the incidence and key characteristics of individuals with liver disease in Wales, using a combination of anonymized individual-level, linkable population-scale primary, secondary, and mortality data. This study was undertaken through a partnership between NHS hepatologists (Pembroke/Yeoman), Cardiff University primary care epidemiologists (Ahmed/Davies), and Swansea University data scientists (Akbari/Gao).

The Wales Liver Disease Registry methodology is based on the International Classification of Diseases (ICD) version 10 (ICD-10) code mapping in the HepaHealth project ( https://easl.eu/publication/hepahealth-project-report/), a recent European-wide epidemiological evaluation of risk factors and preventive actions ^3^ and a 2021 expert panel consensus document ^4^. Codes reflecting the natural history of liver disease have previously been defined to enable monitoring of disease progression over time from underlying etiology (i.e., alcohol-related liver disease, hepatitis C infection) to cirrhosis, portal hypertension, decompensation, liver cancer, and death ^5^.

In this population-based observational study, individuals diagnosed with liver disease during our study period (01/01/2004 to 31/12/2022) were identified using the Secure Anonymized Information Linkage (SAIL) Databank, which incorporates Welsh Longitudinal General Practice (WLGP) primary care data, Patient Episode Database for Wales (PEDW) secondary care data, and Annual District Death Extract (ADDE) from the Office for National Statistics (ONS) mortality data. The SAIL Databank is one of the richest anonymized individual-level, population-scale trusted research environments (TRE) in the world, containing a variety of health and social care, administrative, geo-environmental, and specialist data sources (saildatabank.com Ford *et al.*, 2009, Rodgers *et al.*, 2012). SAIL contains 86% primary care and 100% secondary care data for all Welsh residents and those receiving NHS Wales services. The wealth of information available in the SAIL Databank includes lifestyle and clinical data (alcohol excess, BMI, injecting drug use, smoking, and diabetes), prescribing data, clinical results extracted directly from laboratory reporting systems (e.g., abnormal liver function tests and thrombocytopenia), and environmental and socio-economic deprivation data. ICD-10 codes were mapped to read codes to capture primary SAIL with secondary care diagnoses of liver disease. Liver disease diagnoses were drawn from three 3 sources; secondary care (PEDW), primary care (WLGP), and death certification (ADDE). The overlap between these three sources is displayed in a Venn diagram ( Figure 1). Discrete prediagnosis stages of liver risk factors, alcohol consumption, obesity, and metabolic syndrome diagnoses were defined to enable assessment of the progression of liver disease in a further extension of the Liver Disease Registry methodology.

### Outputs from Liver Research Cymru SAIL Partnership

1) The first interrogation of this RRDA was to describe key clinical and sociodemographic features associated with liver disease diagnosis, later-stage presentation, mortality on first hospital admission, and disease progression.

**Figure 2.**
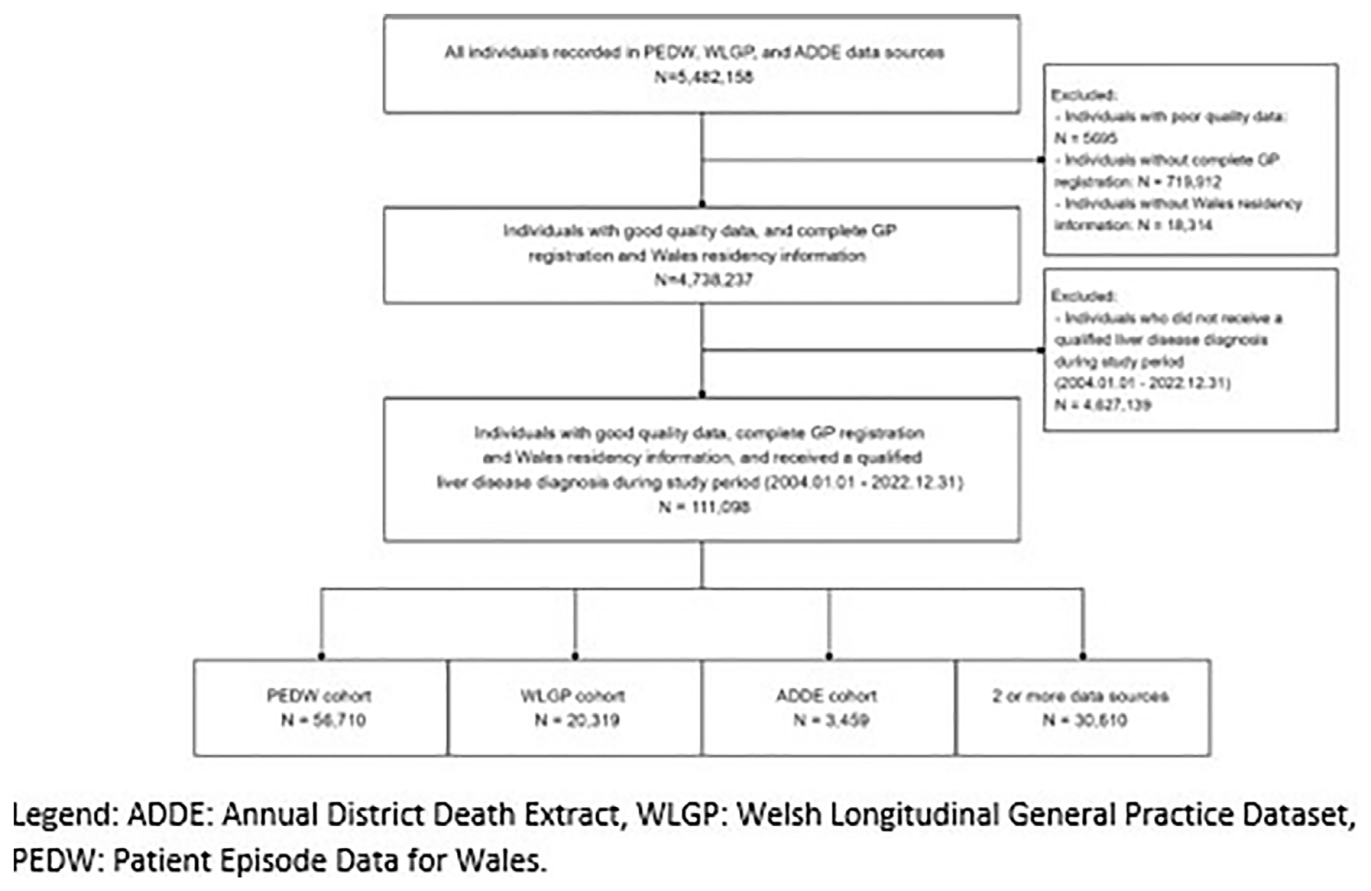
The selection of participants from PEDW, WLGP and ADDE data.

Figure 2 shows the selection of participants from the PEDW, WLGP, and ADDE data sources.

**Figure 3.**
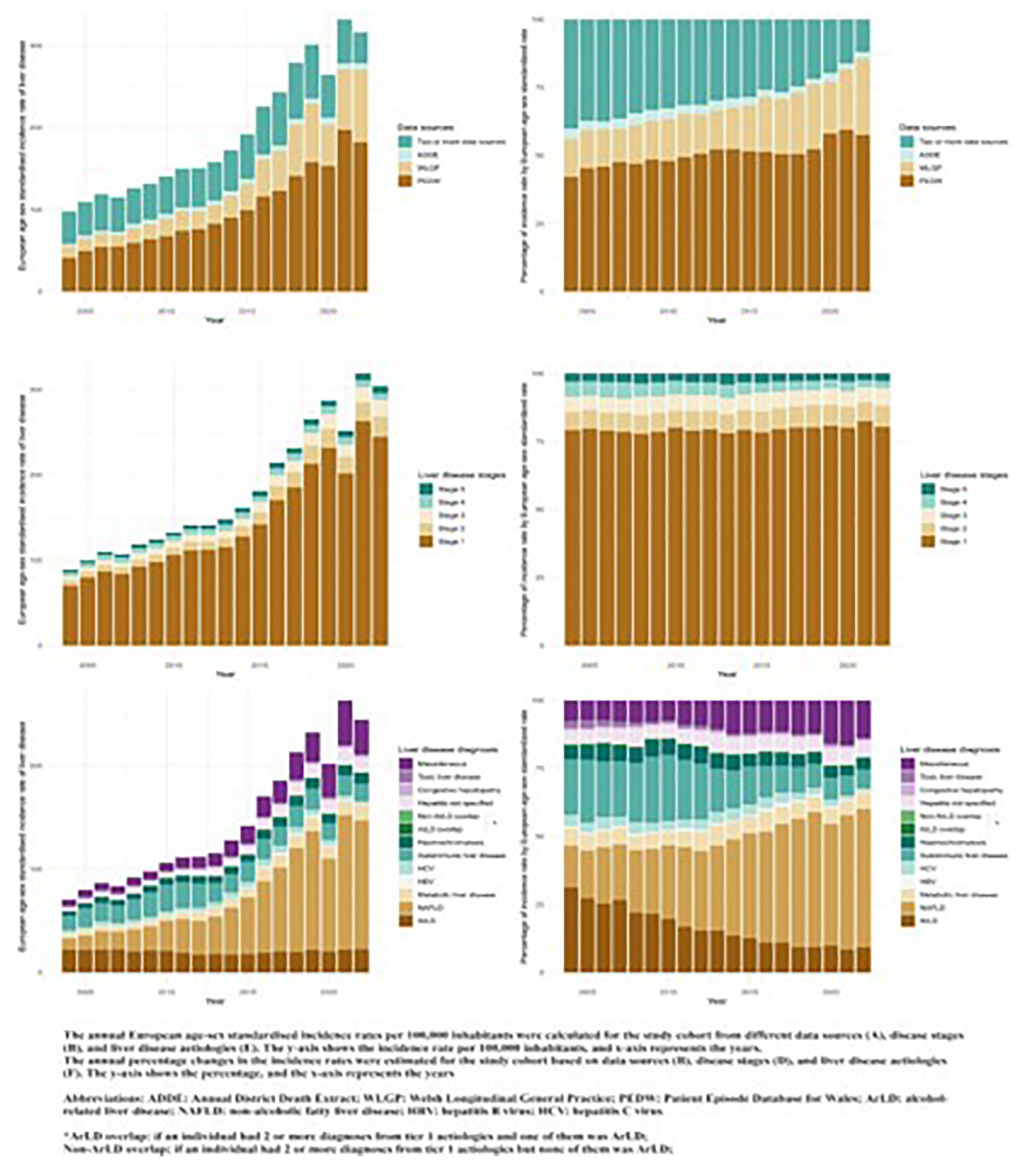
Annual EASR Incidence broken down by data source, disease stages and liver disease aetiology.

Between 2004 and 2022, 111,098 individuals diagnosed with liver disease in Wales were included in this study. The incidence of liver disease increased three-fold during the study period (2004:97.7 per 100,000 individuals in 2004; 2022:316.2/100,000 individuals). A total of 79,992 individuals (72%) entered the cohort with the etiologies of liver disease, including alcohol-related liver disease (ArLD), non-alcoholic fatty liver disease (NAFLD), viral hepatitis, metabolic, hemochromatosis, and autoimmune liver diseases. NAFLD contributed to most of the change in incidence, with an eleven-fold increase during the study period (2004:10.8 per 100,000 individuals; 2022:120.4 per 100,000 individuals, Figure 3). We observed a high proportion of individuals with onset comorbidities, such as hypertension (24,852 [23.7%]), diabetes (3,811[3.6%]), and CVD-related conditions (4,998[4.8%]), over 10 years prior to their first liver disease diagnosis. Furthermore, we noticed an increasing number of incident cases identified from primary care data during the past decades (14% in 2004 and 28% in 2022). This work is currently being submitted for review by the European Journal of Epidemiology. The results are shown in Figure 3.

2) We are currently finalizing a second study protocol to further investigate changes in the frequency of primary care diagnoses at an earlier stage of disease progression. In particular, we define the ability of data assets to assess the impact of service improvement projects on a national scale. We are refining our ability to do this by evaluating the abnormal ALL LFT pathway. This pathway was implemented in a staggered manner, allowing for the analysis of intervention and comparator groups using data from the SAIL Databank across the University Health Boards in Wales. This analysis will include the clinical and cost-effectiveness outcomes. It is anticipated that this analysis of liver health in Wales will identify gaps in service provision and need for service development. These potential low-hanging opportunities to bring about rapid and high-impact service development will be implemented through the NHS Wales Liver Disease Implementation Network. Following this work, we will develop procedures to enable rapid clinical and economic analyses of future pathways, not just in Wales, but across the UK, using routinely collected data and SAIL as the trusted research environment.

(3) We are currently developing protocols to assess the time to diagnosis and disease progression. Linked primary care, secondary care, laboratory, pathology, and mortality data will allow us to describe the clinical encounters from a first presentation with a liver-related symptom to the first liver disease diagnosis and assess the potential for earlier diagnosis and treatment, as well as resource use and associated costs. The main objectives of this work will be descriptive, aiming to better understand the data, size, complexity, and value of repeated measures, and to draw on expertise from other disease areas to define parameters of interest (e.g., code lists of agreed liver-related symptoms, the period that most accurately reflects time to diagnosis). We will use causal inference methods to fully and robustly understand pathways for diagnosis and treatment and identify areas for intervention.

Descriptive logistic regression models investigate a number of potential explanatory variables, including (but not limited to) social deprivation and difficulty in reaching geographic locations (coastal and post-industrial towns and inner cities). The description of features associated with the development of compensated advanced chronic liver disease (cACLD) is of particular interest. These patients are a key group for early diagnosis in primary care, and these analyses will improve our understanding of disease progression, (including HCC development) and potentially lead to future research on appropriate screening strategies. Overall, these descriptive analyses will help us to better understand who is at greater risk of liver disease diagnoses and liver disease-related poor outcomes and will inform subsequent funding applications to allow targeting of the population most at need.

This data analysis will enhance our understanding of the natural history of liver disease and the cost of illness in Wales. We aimed to develop liver disease progression and features of mortality data relevant to under-researched areas, including palliative care and networks for advanced complex liver disease outside the liver transplant setting. Approaches to identifying the risk factors for missed opportunities in diagnosis will be identified.

### Work Programme 3: Prioritising and designing studies

Work packages 1 and 2 delivered the necessary objectives to support work package 3 to lay the foundations for research prioritization and future research delivery.

First, WP 1 built a network of colleagues with different expertise and skills required to deliver liver disease research in Wales.

Second, WP 2 used population-scale multi-source data to create a highly characterized e-cohort of people in Wales with liver disease. To date, this RRDA has been used to describe the current landscape of liver disease in Wales, including the prevalence and incidence of liver disease, socio-demographic characteristics of this population, and a better understanding of the etiology of liver disease in Wales over the last 18 years. This work lays the foundation for rapid data analysis to address new, emerging, and priority questions in liver disease research.

### Research prioritisation

To develop and identify research priorities within liver disease, we reflected on the findings from Work Package 2 to identify the key themes and trends. and aligned these with key priorities for clinicians, patients, and clinical guideline bodies using NICE guidance and the James Lind Priority Setting Partnership “Top 10’s.”

In this regard, alcohol related liver disease is recognised as a priority area of research by the James Lind Alliance (JLA) Top 10s of priorities for research | James Lind Alliance (nihr.ac.uk). The Priority Setting Partnership (PSP) with the JLA has identified 10 key priorities for further research on alcohol-related liver disease. Work package 2 by Liver Disease Cymru identified significant increases in the prevalence of liver disease, particularly alcohol- and non-alcohol-related liver disease. Within the NICE Guidelines [NG50] Cirrhosis in over 16s: assessment and diagnosis (2016), transient elastography (Fibroscan) is advised for men drinking over 50 units of alcohol per week and women drinking over 35 units per week. At present, this is an underutilized resource, and as a non-invasive test, it can be used in primary care and community clinics to help identify cirrhosis. We propose to explore the use of transient elastography in people who drink alcohol at or above the level of units specified by NICE in guideline NG50 in community settings, with the aim of identifying its long-term impact on rates of diagnosis and progression to advanced liver disease. Furthermore, we believe that it is important to evaluate the acceptability of an intervention of this nature in the at-risk population to understand the concerns and expectations of this intervention. Therefore, we undertook a qualitative study of the participants exposed to this intervention.

### Involvement of stakeholders

Work done in WP1 has allowed us to build a network of expertise, including a public and patient perspective, which includes individuals with lived experience of liver disease and charity organization; a clinical perspective, including secondary and primary care; and academic expertise. This network will allow us to strike the right balance and key priorities as a research group to identify and explore further.

### Capacity and capability

Work undertaken in other work packages now provides a platform to allow liver research to be developed through methodological work done within SAIL and delivered from Wales and across the UK. Through this work, we have been able to engage with other research groups across the UK to increase our involvement in and input into liver disease research throughout the UK and to draw potential research projects to Wales. In particular, we have developed strong links with counterparts in Scotland and have been involved in developing a proposal for NIHR funding alongside the Scottish Hepatology Access Research Partnership (SHARP).

software ( https://www.nhsresearchscotland.org.uk/research-areas/hepatology/sharp).

SHARP’s work is focused on the early detection of liver disease, and in particular, on improving access to liver services, which aligns with Liver Disease Cymru’s aims. We propose the development of a tool using routinely collected electronic health records (EHR) and linked data sources, which will help identify the risk of emergency hospitalization for liver disease (of any etiology) in the next 1, 3, and 5 years. This would involve cross-national work with Scottish counterparts and work alongside NHS Glasgow Safe Haven/Robertson Centre for Biostatistics trusted research environments in Scotland and Digital Health Care Wales in Wales. This collaborative approach will allow a robust analysis and evaluation of the effectiveness of interventions across different environments. Furthermore, later work packages will look to determine the acceptability and implementation of this approach with a number of different stakeholders and to evaluate its economic impact.

In addition, we are collaborating with the Nottingham NIHR Liver Disease Research Partnership This group is currently applying for research funding to expand the KLIFAD (Knowledge of Liver Fibrosis in Affects Drinking) project to open a recruitment site in Wales (Aneurin Bevan University Health Board).

Therefore, we anticipate that the work undertaken within the LRC in WP 1 and 2 and the engagement with other funded research partnerships supported by this work will allow the nascent liver research community in Wales to “buddy up” with research colleagues and organizations across Wales that can support our ability to deliver research in this field as well as with more established liver research groups across the UK.

## Summary and conclusion

The NIHR grant provided a strong stimulus to link Hepatologists in Wales with the broader existing research environment across the nation. This process began with drawing together the necessary expertise to submit the grant proposal, continued via the three programmes outlined above, and has created a legacy that we hope to be able to build on.

In the short term, there remains a lack of dedicated research-funded posts in Wales to apply for large-scale grants in isolation; however, LRC partnerships have created a knowledge base and connectivity that allows us to collaborate with other more established academic centers. This is especially so in relation to the SAIL data, where much of the funding was prioritized.

## Ethical consideration

Approval for the use of anonymised data in this study, provisioned within the Secure Anonymised Information Linkage (SAIL) Databank, was granted by an independent Information Governance Review Panel (IGRP) under project 1492. The IGRP has a membership comprised of senior representatives from the British Medical Association (BMA), the National Research Ethics Service (NRES), Public Health Wales and Digital Health and Care Wales (DHCW). The usage of additional data was granted by each respective data owner. The SAIL Databank is compliant with General Data Protection Regulations (GDPR) and the UK Data Protection Act.

All data were accessed via the SAIL Databank trusted research environment (TRE) following approval from the independent Information Governance Review Panel (IGRP). For data held within SAIL under the core-restricted data – additional approvals are sought from their respective data owner as per the data sharing agreement (DSA) in place with the data owners and SAIL. For our project have approval to the WCSU (Welsh Cancer Intelligence Surveillance Unit - WCISU) and ICNC (Intensive Care National Audit & Research Centre - ICNARC) which were approved by Public Health Wales and ICNARC respectively via the IGRP application and approvals process, under the same legal basis as all other data accessed in SAIL by the project. For more information on the SAIL Databank please see https://saildatabank.com/

## Data Availability

The data used in this study are available in the SAIL Databank at Swansea University, Swansea, UK, but as restrictions apply, they are not publicly available. All proposals to use SAIL data are subject to review by an independent Information Governance Review Panel (IGRP). Before any data can be accessed, approval must be given by the IGRP. The IGRP carefully considers each project to ensure the proper and appropriate use of SAIL data. When access has been granted, it is gained through a privacy-protecting trusted research environment (TRE) and remote access system referred to as the SAIL Gateway. SAIL has established an application process to be followed by anyone who would like to access data via SAIL at https://www.saildatabank.com/application-process.

## References

[1] Pembroke TPI John G Puyk B : Rising incidence, progression and changing patterns of liver disease in Wales 1999–2019. *World J Hepatol.* 2023;15(1):89–106. 10.4254/wjh.v15.i1.89 36744166 PMC9896508

[2] Macpherson I Abeysekera KWM Harris R : Identification of liver disease: why and how? *Frontline Gastroenterol.* 2022;13(5):367–373. 10.1136/flgastro-2021-101833 36051960 PMC9380769

[3] Abeysekera KWM Macpherson I Glyn-Owen K : Community pathways for the early detection and risk stratification of chronic liver disease: a narrative systematic review. *Lancet Gastroenterol Hepatol.* 2022;7(8):770–780. 10.1016/S2468-1253(22)00020-6 35525248

[4] Pimpin L Cortez-Pinto H Negro F : Burden of liver disease in Europe: epidemiology and analysis of risk factors to identify prevention policies. *J Hepatol.* 2018;69(3):718–735. 10.1016/j.jhep.2018.05.011 29777749

[5] Hagström H Adams LA Allen AM : Administrative coding in Electronic Health Care Record-Based research of NAFLD: an expert panel consensus statement. *Hepatology.* 2021;74(1):474–482. 10.1002/hep.31726 33486773 PMC8515502

