# Supplementary material for: The development of Liver Research Cymru, a new partnership to increase hepatology research activity in Wales
Source: NIHR Open Res. 2024 Oct 8;4:57. [Version 1] doi: 10.3310/nihropenres.13663.1 (PMC12421226; doi:10.3310/nihropenres.13663.1)
Supplement: Peer review history

# Reviewer report: Reviewer response for version 1

**DOI:** 10.3310/nihropenres.14834.r33212

**Published:** 2024-11-25

**Authors:** Jee-Fu Huang

**Affiliations:** 1 Kaohsiung Medical University, Kaohsiung, Taiwan

The study aimed to assess the efficacy of  a multi-disciplinary collaboration work in tackling the barriers for early detection of HCC in Wales. Three directions of strategy were developed and designed in the study. Some of the patient reported outcome were measured with questionnaires in addition to the collection of the epidemiological features. Other measurements of each direction of the strategy were depicted in a precise manner. The study added the current important issue for early detection and disease awareness of the high mortality cancer, if not detected in an early stage. Some points need clarification.

 1. The major causes of HCC are viral hepatitis infections such as HBV and HCV. The grade of disease awareness between different viral etiology may vary. Also the hepatocarcinogenesis of different viral etiology could be different. The balance approaches could be adjusted.

 2. The early detection in a large part depends on the facility and capacity of the medical institutes. The difference of early detection could be stratified in different hospital level.

 3. Most of the HCC are asymptomatic in a early stage. How to overcome the barrier based on patient's educational and socioeconomic levels?

Is the work clearly and accurately presented and does it cite the current literature?

Yes

If applicable, is the statistical analysis and its interpretation appropriate?

Yes

Are all the source data underlying the results available to ensure full reproducibility?

Yes

Is the study design appropriate and is the work technically sound?

Yes

Are the conclusions drawn adequately supported by the results?

Yes

Are sufficient details of methods and analysis provided to allow replication by others?

Partly

Reviewer Expertise:

Hepatology, Epidemiology, Fatty liver disease, Liver cancers

I confirm that I have read this submission and believe that I have an appropriate level of expertise to confirm that it is of an acceptable scientific standard, however I have significant reservations, as outlined above.

# Response

**Published:** 2025-01-10

**Authors:** andrew yeoman

**Affiliations:** Gwent Liver UNit, NHS Wales Aneurin Bevan University Health Board, Newport, Wales, UK

Hi,

 Our report is in relation to building a collaboration to improve liver disease research in Wales not in relation to HCC detection

 Do these comments relate to a different study?

 Best wishes

 Andrew Yeoman

# Reviewer report: Reviewer response for version 1

**DOI:** 10.3310/nihropenres.14834.r33214

**Published:** 2024-11-15

**Authors:** Sofia Hemrage

**Affiliations:** 1 King's College London, London, England, UK

Thank you for your report on the Liver Research Cymru, a new partnership to increase hepatology research activity in Wales. I read with interest this article on LRC, focusing on an emerging, much-needed research area. The article provides a thorough account of LRC, aimed at advancing current research in the area. The scope of LRC appears promising to advance prevention, detection, care delivery and research. Each work programme is described, and respective objectives and methods are clearly explained.

 Please find below a list of queries and suggestions:

1. The work is clearly presented – however, there are a few typos and reference formatting issues (i.e. style, and a few odd links).
2. **Introduction: **It would be useful to specify and quantify the burden of liver disease: are the authors referring to admissions? Years lost to disability? Mortality? Re-hospitalisation rates? This would provide a more comprehensive understanding of the magnitude of the burden of liver disease in Wales, and therefore further illustrate the relevance of the partnership.
3. The overall work would benefit from including additional literature to better support the arguments presented.
4. As one of the main aims of the LRC is to develop novel ways to tackle the deprivation gradient, the work must be conducted so that it is relevant to people from underserved groups. I appreciate that the authors conducted PPI to inform the design and involved people with individual experiences from the early stages of the research lifecycle. In the interests of validity and transparency, it would be useful to include demographic information of the involved members of the public or expand on/acknowledge any efforts made to reach a range of people that ensure the validity and inclusivity of the research (although this is later covered in page 5). Some information is provided in page 8, however it could be useful to have additional detail in aspects including ethnicity, education attainment and housing status.
5. There is a substantial amount of qualitative literature describing the experiences of research nurses in managing care delivery expectations and research performance. This particular group of the workforce, who is often expected to deliver care as a frontline member while meeting research targets and ensuring optimal performance, is often met with barriers including role conflict. Given their central role in research delivery, it would be useful to know whether efforts were/will made to include their expertise during engagement work, to better inform on the feasibility of future research. This can foster participatory research approaches and a more egalitarian, sustainable research culture.
6.  In the case of ARLD, a major barrier to effective care is the lack of liver training in community treatment settings. It is also in this setting where prevention, early detection and intervention would be most useful. Therefore, it would be useful to understand to what extent the research team involved healthcare providers from the third sector in the work, and if there are plans to include those in the LRC.
7. The quality of the figures makes them challenging to understand.

The points listed above are more out of interest/transparency and on a suggestion basis – I do not anticipate major revisions being necessary. I look forward to reading the future work of the LRC.

Is the work clearly and accurately presented and does it cite the current literature?

Partly

If applicable, is the statistical analysis and its interpretation appropriate?

Not applicable

Are all the source data underlying the results available to ensure full reproducibility?

No source data required

Is the study design appropriate and is the work technically sound?

Yes

Are the conclusions drawn adequately supported by the results?

Yes

Are sufficient details of methods and analysis provided to allow replication by others?

Yes

Reviewer Expertise:

Applied health research, alcohol-related liver disease, psychosocial interventions, health inequalities, mixed-methods research

I confirm that I have read this submission and believe that I have an appropriate level of expertise to confirm that it is of an acceptable scientific standard, however I have significant reservations, as outlined above.

# Response

**Published:** 2025-02-04

**Authors:** andrew yeoman

**Affiliations:** Gwent Liver UNit, NHS Wales Aneurin Bevan University Health Board, Newport, Wales, UK

Authors response

 We thank the reviewer for their comments

 Point 1. We have reviewed and amended the manuscript where typos exist

 Point 2. The burden we describe relates to incidence and severity of liver disease. This is based on existing coded data published as cited in this report (reference 1). WE did not expand on that in the interests of keeping the word count down. In terms of severity this is supported within that same publication by significant increase not just in coded liver disease (which might merely represent better awareness) but in Hepatocellular cancer, Cirrhosis and Portal Hypertension and admissions and mortality .

 I have expanded the introduction to reflect this and made additional references 

 Point 3. Additional literature has been added in introduction and in later text regarding research priorities and that we have now had work from WP 2 accepted for publication

 Point 4. PPI group members. We did not ask the housing status or educational attainment of the PPI members. We did attempt to capture a broad range of to cover different liver diseases and a geographical split across Wales. We did this by working with the British Liver Trust who assisted us in recruiting people. Every member was white – 2021 census data shows that 94% of the Welsh population identifies as such. This remains an important issue however and we remain keen to recruit people of different ethnic backgrounds. Since the development of this collaboration we have developed informal links with Muslim Doctors Cymru who we can use as a conduit to try and recruit other PPI members in the future. WE have added a comment in the discussion addressing the limitations within the recruited PPI group.

 Point 5. The research infrastructure with the NHS in Wales is significantly different to England and we do not have dedicated liver research nurses. We do not dispute the impact and importance of their role but as we have none currently it was not possible to bring their expertise to inform our approach. If we are able to develop this critical workforce over time then clearly we would utilise their expertise in any planned future research.

 Point 6. We did not involve 3rd sector services in the original collaboration. However one of the early potential collaborations that have come out of LRC is in relation to a project evaluating the utility of Fibroscan to bring about behaviour change in relation to ARLD. This would be delivered within community 3rd sector delivered settings. The preliminary discussions will hopefully allow us to bring this additional expertise into our collaborative.

 Point 7. Apologies for this. Higher resolution images have been uploaded.
